# Influence of Gait Cycle Loads on Stress Distribution at The Residual Limb/Socket Interface of Transfemoral Amputees: A Finite Element Analysis

**DOI:** 10.1038/s41598-020-61915-1

**Published:** 2020-03-19

**Authors:** Sofía C. Henao, Camila Orozco, Juan Ramírez

**Affiliations:** Department of Mechanical Engineering, Universidad Nacional de Colombia, Medellin, Colombia

**Keywords:** Quality of life, Orthopaedics, Disability, Pain, Biomedical engineering

## Abstract

A Finite Element Analysis (FEA) was performed to evaluate the interaction between residual limb and socket when considering the dynamic loads of the gait cycle. Fourteen transfemoral amputees participated in this study, where their residual limbs (*i.e*., soft tissues and bone), and their sockets were reconstructed. The socket and the femur were defined as elastic materials, while the bulk soft tissues were defined as a hyperelastic material. Each model included the donning, standing, and gait cycle phase, with load and boundary conditions applied accordingly. The influence of adding the dynamic loads related to the gait cycle were compared against the modelling of the static load equivalent to the standing position resulting in changes of 23% ± 19% in the maximum values and in an increase in the size of the regions where they were located. Additionally, the possible correspondence between comfort and the location of peak loadbearing at the residual-limb/socket interface was explored. Consequently, the comfort perceived by the patient could be estimated based on the locations of the maximum stresses (*i.e*., if they coincide with the pressure tolerant or sensitive regions of the residual limb).

## Introduction

In lower limb amputees, specifically for limb amputations above knee level, the prosthetic system consists mainly of a prosthetic foot, a prosthetic knee and a socket. Since the attachment between the residual limb and the prosthesis occurs through the socket, its coupling is critical for patients looking to regain their functional mobility and perceiving comfort^1,2^. Among others, the main task of the socket is to distribute the loads applied on the residual-limb/socket interface. By doing this, the stresses generated at the residual limb in sensitive areas are reduced, and gait becomes stable by allowing adequate proprioception and movement of the residual limb muscles^3–5^ (all of this during a prolonged loading time^6^). Thus, the residual-limb/socket interface is a key factor in the successful performance of the patient during his/her rehabilitation^7^.

When wearing a prosthesis, both normal (*i.e*., perpendicular to the skin) and shear stresses (*i.e*., tangential to the skin) are applied to the soft tissues of the residual limb, which are not accustomed to bear such elevated loads, inducing the risk of skin problems and chronic pain^1,2,6^. Recommendations for transtibial socket design based on Pain Pressure Thresholds (PPT) have been defined by other authors since pressure beyond certain limit triggers pain^8,9^ and pressure is the condition that most critically increases skin problem and chronic pain risks at the residual-limb/socket interface^10^. Moreover, the existence of algometers as reliable medical devices for measuring PPT^11,12^ can lead to easier implementation of new protocols for socket design where pressure tolerant and sensitive regions at the residual limb are defined according to the PPT that patients can endure based on their perception^2,6,13,14^. These thresholds are established when critical stresses and strains states exceed tolerable values. However, these thresholds may increase with larger contact areas or when the load is applied intermittently^4,15^. In addition to these pain thresholds, pressure values have been reported at which capillary vessels are unable to conduct blood (from 30 *mmHg* (4 *kPa*)^16^ to 60 *mmHg* (8 *kPa*)^4^), being these pressure values critical for muscle tissues due to their greater vascularity and their metabolic demands (it should be noted that this injury pressure levels are substantially smaller than the pressure tolerance levels reported in this research). A successful socket design must relief pressure from sensitive regions and exert pressure on tolerant ones. Nevertheless, current socket design and manufacture are based on the prosthetist’s ability and professional experience rather than on quantitative data^7^.

FEA has been used as an instrument for evaluating a wide range of aspects of lower limb amputees. FEA allows a less subjective analysis of factors that are difficult to study analytically or measure experimentally, including strains and stresses generated at the residual-limb/socket interface during the use of the prosthesis^1–3,10,14,17–19^.

Although FEA on transfemoral amputees have been studied before, there is a need to evaluate the behaviour of the interface under dynamic loads. This will allow to analyse higher precision models for residual-limb load and boundary conditions that do not ignore changes in the coupling between the socket and the lower limb due to relative angular movements or axial displacements, or changes of variations in velocity and acceleration^1^. These dynamic conditions are present during the gait cycle, which is affected by the endogenous state of the amputee given that the asymmetry of the gait during ambulation radically differs from healthy gait parameters. Additionally, when individuals try to return to their dynamic stability, various compensations in forces and moments are generated that result in specific pathologies affecting the load distribution on the residual limb. Dynamic models that successfully include these compensated forces and moments could lead to more precise simulations. The aforementioned will not be evident in models that only take into account the static loads caused by the amputee’s weight^19–21^ which, to the best of the knowledge of the authors, are the only loads currently reported for transfemoral amputees^1^.

This study aims to analyse the stress distribution of the residual limb of 14 transfemoral amputees, evaluating which zones are highly exposed to pressure and shear stresses based on FE results. Additionally, the maximum stresses values and their location, obtained during the gait cycle, are compared with those of a static-load simulation (*i.e*., standing) to identify additional information derived from the dynamic models. Further, the study compares the perception of comfort and the location of the peak loads (*i.e*., feeling comfortable when the peak loads are located at pressure tolerant regions or feeling uncomfortable when they are located at pressure-sensitive regions).

## Methods

Fourteen unilateral transfemoral amputees participated in a series of experiments. Before the procedure, they all signed an informed consent bearing all pertaining information and written in accordance with the guidelines set by the Ethics Committee. All experimental protocols were approved by the Ethics Committee of Universidad Nacional de Colombia (CEMED-145-10) and the experiments were carried out in observance of the World Medical Association’s Code of Ethics and the Declaration of Helsinki.

Table 1 shows the general amputation-related information of each subject and the data and variables used to analyse the results. Most participants used their prosthesis throughout the day and lived a fairly active lifestyle. They had no other physical, vascular, neurological, or psychological condition that may alter or modify the results of the numerical model simulation.

**Table 1:** Amputees’ general information.

Patient	Time since amputation [years]	Socket type	Weight without Prosthesis [Kg]	Prosthesis Weight [Kg]	Estimated Weight [Kg]	Height [cm]	Leg Length [cm]	Residual limb length [cm]	Body mass index BMI_E_ [kg/m2]
P01	9	Quadrilateral	72	5	82,5	176	86,5	28	26,6
P02	2	Quadrilateral	57	4,1	65,8	172	80,5	21	22,2
P03	7	Quadrilateral	91,5	4,9	105,5	177	86	23	33,7
P04	1	Quadrilateral	83,8	4,9	94,5	165	74	34	34,7
P05	2	Semi ischial- containment	75,3	2,9	84,8	168	76,5	36,5	30,0
P06	4	Semi ischial- containment	67,9	3,2	77,5	175	85	30	25,3
P07	24	Exomodular	73,6	6,2	85,0	167	79	20	30,5
P08	13	Semi ischial- containment	49,1	4,1	56,5	153	69,5	20,5	24,1
P09	6	Quadrilateral	72	4,1	83,3	167	80	19	29,9
P10	42	Semi ischial- containment	64,5	3,7	75,6	161	77	10	29,2
P11	23	Quadrilateral	64,7	2,8	74,0	164	83	28	27,5
P12	4	Quadrilateral	81	6,6	92,4	171	85	31	31,6
P13	3	Quadrilateral	59	4,6	68,4	162	78	17	26,1
P14	5	Semi ischial- containment	59	4	67,9	168	83	24	24,1

Regarding *BMI*, the method created by Mozumdar and Roy^22^ was used to calculate the estimated weight (*W*_*E*_). This method infers the *W*_*E*_ from the current body weight, and its reduction as a consequence of the amputation (Δ*W*). The fraction of weight lost due to the missing limb was defined according to the values reported by Osterkamp^23^ for different body parts, and the proportion of the thigh that was amputated, which was calculated during the experiments. The information used for the calculation and the *BMI*_*E*_ results for each patient are shown in Table 1.

**Figure 1:**
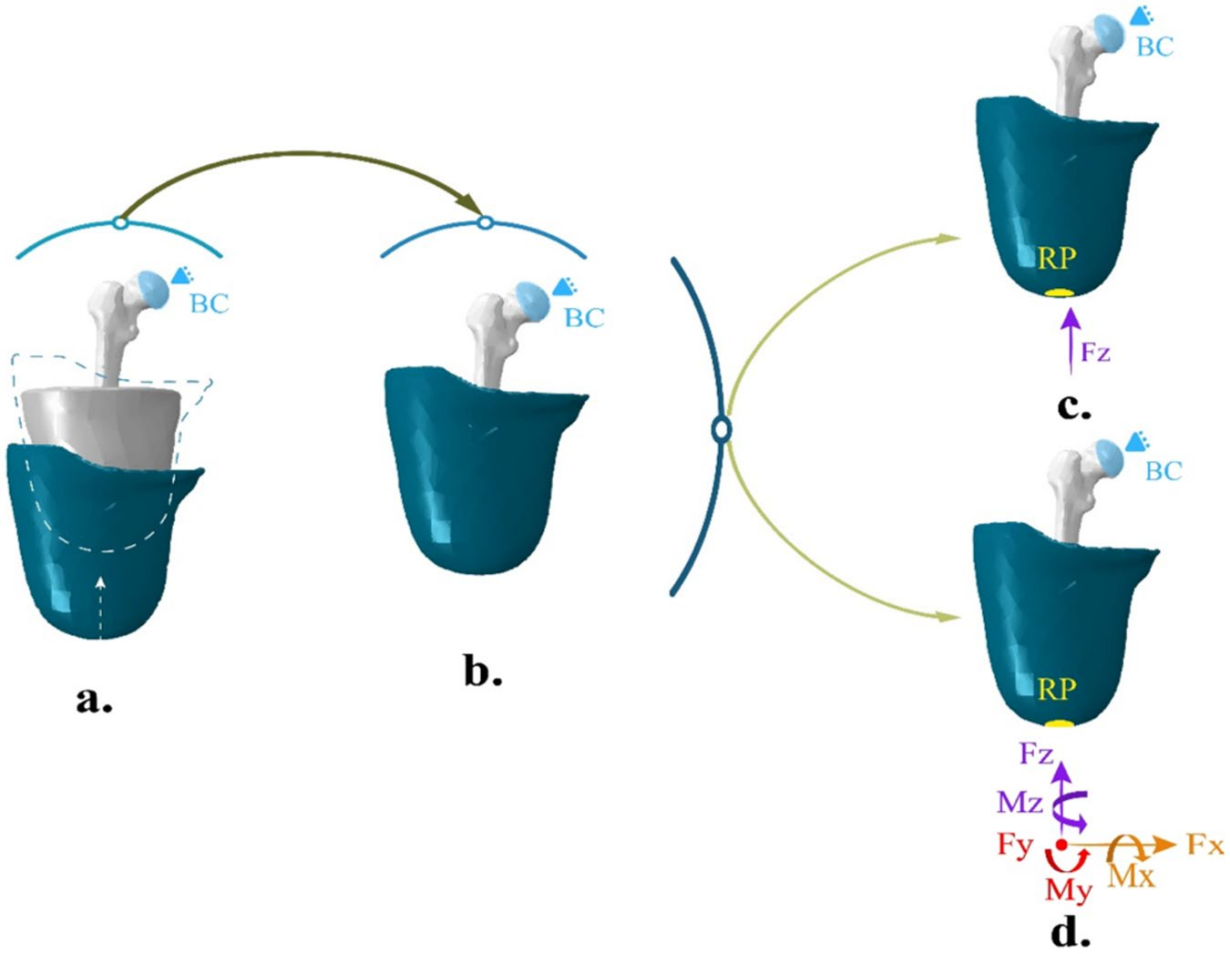
Numerical simulation sequence: (**a**) Socket donning: displacement of the socket to its final position and solution of initial overclosure; (**b**) Stabilization: created to mitigate the effects of the socket donning and to allow the soft tissues to acquire their final position; (**c**) Static load condition: simulation of the standing position; and (**d**) Dynamic load condition: simulation of the complete gait cycle. Where *BC* stands for the representation of the hip joint action restricting all degrees of freedom relative to displacement; *RP* is the zone where all the loads are applied, coinciding with the coupling of the prosthetic device and the socket; and each arrow represents the anatomical direction of the Forces (*F*) and Moments (*M*) simulating the corresponding load condition.

Figure 1 shows the simulation process. The load and boundary conditions of each phase were applied accordingly to faithfully simulate the use of the socket from the donning to the gait cycle. The numerical model was carried out in three stages: socket donning (a), a period of stabilization (b) and the load phase. This final phase includes the model of the static condition (*i.e*., standing position) (c) and the dynamic condition (*i.e*., complete gait cycle) (d).

### Geometry

During socket design, as a general rule, the prosthetist aims to achieve an adequate load distribution by compressing tolerant regions in the residual limb and relieving pressure from intolerant regions to make the socket more comfortable for the amputee^8,24^. In this study, the socket and the residual limb were digitally reconstructed as separate elements using a 3D laser scanner (NextEngine Inc., Santa Monica, USA). The socket was obtained by scanning a rectified positive plaster while the external surface of the residuum soft tissue was generated from a direct positive plaster cast of the residual limb. Although surgical scars played an important role in numerical models^25^, they were purposely excluded in this study because the main objective was to simulate the interaction between the socket and the residual limb. Additionally, including the surgical scars would complicate the solution of the FEM model without adding valuable information to the volumetric response of the residual limb. The femur of the amputee was also included in the 3D reconstruction of the residual limb using information from a computerized axial tomography (CT) to define its geometry and relative position to the soft tissues. To appropriately define the relative position of the three elements, marks were made to indicate the location of the greater trochanter and the ischial tuberosity in the 3D scanned parts. Finally, the CT information was used to align the residual femur with the soft tissues and the socket.

Detailed information on the tomography parameters, geometry reconstruction, and assembly and donning procedures followed in this study can be found in Lacroix and Ramirez^26^.

### Materials

The material’s mechanical properties (*i.e*., soft tissue, bone and polypropylene) were not custom-tailored for each patient. All sockets and bones were considered a linear elastic homogeneous isotropic material and all soft tissues, a hyperelastic material. The properties were in the same order of magnitude as those used by other authors, although those studies mainly focused on the socket/residual-limb interaction for transtibial amputees^19,25,27–33^. For the bone, Young’s modulus was set to 15 *GPa*, Poisson’s ratio to 0.3 and density to 2000 *kg*/*m*^3^ ^34^. As for the socket, which was made out of polypropylene, Young’s modulus was set to 1.5 *GPa*, Poisson’s ratio to 0.3 and density to 800 *kg*/*m*^3^ ^27,28,35^. The software’s defined default values for linear and quadratic bulk viscosity parameters were used (0.06 and 1.2, respectively).

Due to the large strains to which the soft tissues were subjected, a hyperelastic model was selected for its simulation. The behaviour was defined using the following generalized Neo-Hookean strain energy potential equation:

1 $$W={C}_{10}(\overline{{I}_{1}}-3)+\frac{1}{{D}_{1}}{(J-1)}^{2}$$

where the invariants of the principal stretch ratios were ${I}_{1}={\lambda }_{1}^{-2}+{\lambda }_{2}^{-2}+{\lambda }_{3}^{-2}$. The relative volume change was *J*, and *C*_10_ and *D*_1_ were the constitutive parameters. For this study, ${C}_{10}=11.6\,{\rm{kPa}}$ and ${D}_{1}=11.9\,{{\rm{MPa}}}^{-1}$ were identified from an inverse method developed by Affagard *et al*. when assessing a displacement field measurement^36^. Finally, the soft tissues density was set to $1000\,\,kg/{m}^{3}$.

### Boundary conditions

For all the phases, the interaction between the bone and the bulk representation of the residual soft tissues were modelled using a tie condition that simulates perfect bonding between these two elements. The interaction between the socket and the residual limb was modelled using a surface to surface contact condition in ABAQUS V6.12-3 (Dassault Systèmes SolidWorks Corp., Waltham, USA), which prevents the residual limb nodes (*i.e*., slave nodes) from penetrating the socket (*i.e*., master surface) during their relative displacement. The hip joint state was recreated with a restriction on all degrees of freedom relative to the displacements of the femoral head, specifically over the zone where the acetabulum encloses the femur. Additionally, the pressure and shear stresses at the residual-limb/socket interface were caulated.

A dynamic model was de.eloped. Due to the complexity of its geometry, tetrahedral elements were used for all parts. The approximated global sizes of the elements (intern-nodal spacing) were determined after a mesh sensitivity analysis and defined as 5 *mm* for the residual limb and 3 *mm* for the bone and the socket. The number of elements varied between approximately 300.000 and 480.000, depending on the size of the residual limb.

Depending on the patient, the routine lasted between 9 to 18 hours using a Xeon E5-1650 v2 Processor with 64GB RAM on a 64-Bit Windows 10.

#### Socket donning

Considering that during donning there is hair but no sweat at the residual-limb/socket interface, a 0.37 coefficient of friction was assigned to the interaction between the two surfaces^37^. In the simulation, a displacement vector was applied on the distal end of the socket. The value of each vector was equivalent to the displacement needed to situate the residual limb on its actual final position inside the socket from a non-contact position. This usually implied a displacement vector with components in the three axes. Vectors were different for each subject and calculated according to the CT information and the marks on the socket and the residual-limb positive cast. The donning procedure was stopped at the matching of the marks. The condition of displacement was applied to the socket in a quasi-static step requiring the lowest possible velocity to minimize dynamic effects but reasonable time for calculation. As a consequence, although amputees needed only a short time to set their residual limb inside the socket according to the actual donning procedure, the duration of the model for this study was set to 15 *s* and, depending on the condition of socket displacement for each amputee, the velocities varied from 6 to 9 *mm*/*s*.

#### Stabilization

In this intermediate step, no additional stimulus was applied. The dynamic effects of the socket donning were mitigated, and the soft tissues reached their final position. A stabilization time of 5 *s* waset to let the soft tissues adjust to the socket’s interior and reach a position of greater equilibrium.

#### Standing position

The stress states obtained from the previous step were maintained the same while applying a force representing the standing load’s condition. The load was vertically applied to the socket with an equivalent magnitude of half the weight of the amputee. Regarding the coefficient of friction, a change in the dryness of the interface (since sweat was expected to be present) resulted in a new value, 0.23 at the residual-limb/socket interface^37^.

#### Gait cycle

A previous study^38^ was carried out to predict the loads generated at the base of the socket during gait in transfemoral amputees. The data was generated by recording the subject’s whole gait cycle in a motion capture analysis laboratory, using a force plate on a flat surface at natural stride speed. The forces and moments at the joint between the socket and the femoral extension were calculated based on a Multibody System Inverse Dynamics (MSID) approach. This study provided the forces and moment curves at the socket distal end of a reference amputee without major difficulties to walk using an aluminium articulated foot and a pneumatic knee.

Since carrying out the gait analysis for all of the subjects was not possible due to the availability of resources and the time and willingness of the subjects, the 14 amputees’ gait curves were approximated. The gait force and moment curves of the reference amputee were scaled for each of the 13 remaining subjects taking into account that the ground reaction forces and its increment are proportional to the Body Mass Index (*BMI*) during the gait cycle, and that the joint kinematics remain similar^39^. Due to the specific condition of the amputees’ anatomy, the *BMI*_*E*_ (instead of the *BMI*.) of each subject was used as the scale parameter for each curve since it accounts for the missing limb length (the *BMI*_*E*_ calculation procedure and the value for each subject are provided in the Methods section). Although the curve magnitudes were scaled, the shape of the curves was the same for all amputees. Further, it was assumed that the *BMI*_*E*_ takes into account the residual limb length effect at the moments applied at the distal end of the socket, since it is included in the calculation and the lengths do not vary greatly among the subjects (level of amputation of 34% ± 15% and *BMI*_*E*_ = 28.3 ± 3.7).

**Figure 2:**
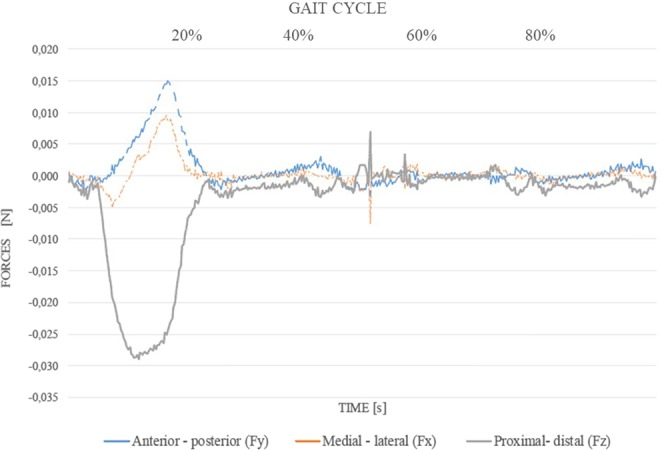
Normalized dynamic loads applied at the socket base for P7. Each line represents forces in the three anatomical directions (medial-lateral, anterior-posterior, and proximal-distal) with magnitude in Newtons (*N*).

The values of forces and moments obtained for each patient were applied at the base of the socket as dynamic loads simulating the complete gait cycle during a time-lapse of 5.26 *s* (which was the actual duration of the movement gesture). Figure 2 shows an example of the gait forces for the reference patient. Each line represents the magnitude of the force in an anatomical direction (anterior-posterior, medial-lateral and proximal-distal) for the femoral extension segment.

**Figure 3:**
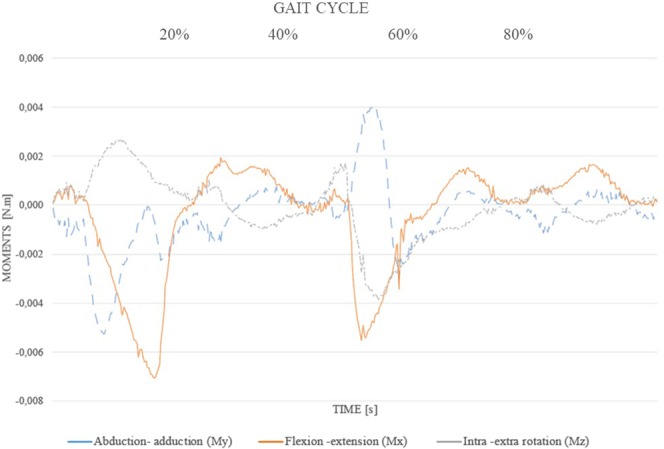
Normalized dynamic loads applied at the socket base for P7. Each line represents moments in the three anatomical directions (flexion-extension, abduction-adduction and intro-extra rotation) with magnitude in Newtons per meter (*N*.*m*).

Figure 3 shows the example of the gait moments for the reference individual. Each line represents the magnitude of the moments that were applied and corresponds to the anatomical direction considered for the femoral extension segment: the rotations in the direction of intro-extra rotation, abduction-adduction and flexo-extension.

The explicit dynamic analysis procedure in ABAQUS was used for the gait cycle. Damping was calculated using the default bulk viscosity values, mass scaling was modified until the model converged, and incrementation time was set as automatic.

**Figure 4:**
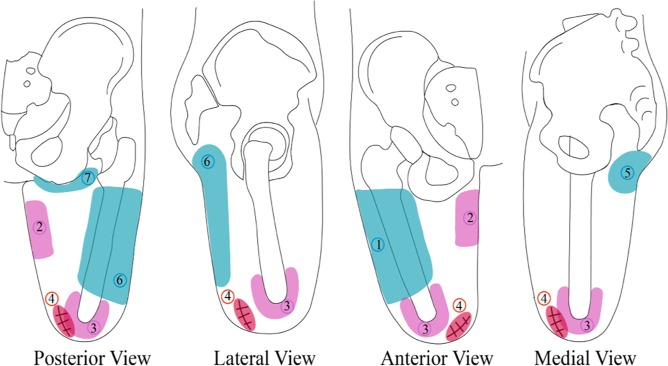
Pressure tolerant (blue) and pressure sensitive (pink and red) areas where: 1: lateral zone from the large abductors and over the femur. 2: medial proximal zone close to the ischium. 3: distal area of the residual femur. 4: scar and its contiguous zones. 5 and 7: gluteus. 6: from the gluteus to the lateral hamstrings.

To analyse the stress distribution, seven zones were obtained with the support of the patients and an experienced prosthetist. The zones are presented in Fig. 4. These zones represent pressure-tolerant and pressure-sensitive areas after the amputation. It is known that a correct fitting, leading to a comfortable prosthesis, depends on the how weight bearing and pressure loads are distributed over pressure-tolerant zones. Peak values of stresses in the residual-limb/socket interaction, and the regions in which they were exhibited were compared for both dynamic and static loading cases.

Additionally, during the acquisition of data for the construction of the numerical models, a comfort test^14^ was carried out to learn whether patients felt comfortable with the use of their prostheses or whether they did not adapt to it completely, hindering their daily life. This test specifically evaluates the comfort sensation on transfemoral amputees, and consist of 30 questions (divided by topic: appearance, well-being, pain, function, psychological factor, and social factor) and, according to a final score, defines if the subject feels or not comfort while wearing the prosthesis. Their responses were analysed according to the location of maximum stresses in the numerical models and the identified zones (*i.e*., pressure tolerant and pressure sensitive regions as shown in Fig. 4). It was expected that amputees would feel comfortable wearing their prostheses if the maximum stresses were located in pressure tolerant regions.

## Results

The analysis focused on the residual-limb/socket interaction. The reaction force magnitude at the acetabulum, at the end of the donning phase, was stated in Table 2. The contact pressure (CPRESS) reported in Table 2 represents the maximum pressure value found at the residual limb’s surface in each phase. For the gait cycle, the maximum value was selected after analysing the results during the whole phase simulation. Regarding the identification of the peak pressure, extreme caution was taken to identify the maximum value within the region supporting highest pressure to prevent false data caused by a compromised element (*e.g*., an element located at the proximal border of the residual-limb reconstruction that does not resemble the actual condition since the residual limb is attached to the rest of the body. This situation increases the stresses values due to concentration factors. Or single elements reporting high stresses without surrounding elements having similar values suggesting meshing problems instead of a true stress distribution). Circumferential (CSHEAR1) and longitudinal peak shear (CSHEAR2) stresses at the time of maximum pressure value were established and are shown in Table 2 (in the table, NN refers to non-needed data because the stabilization phase was not required).

**Table 2:** Reaction forces for the 14 patients present at the acetabulum in the end of the donning phase. Peak values of CPRESS, CSHEAR1 and CSHEAR2 stresses in the stabilization, standing and gait phase. Difference between the Standing and the Gait Cycle (GC-S) values for CPRESS.

Patient	Donning				Stabilization			Standing			Gait cycle			Difference GC-S
	RF [N]	CPRESS [kPa]	CSHEAR1 [kPa]	CSHEAR2 [kPa]	CPRESS [kPa]	CSHEAR1 [kPa]	CSHEAR2 [kPa]	CPRESS [kPa]	CSHEAR1 [kPa]	CSHEAR2 [kPa]	CPRESS [kPa]	CSHEAR1 [kPa]	CSHEAR2 [kPa]	
P1	144	54	−14	−20	120	−14	−23	130	43	36	200	27	37	54%
P2	365	140	28	−48	150	14	20	110	25	38	110	−12	−25	0%
P3	159	5	0	−1	140	−18	−46	180	51	56	190	−28	33	6%
P4	237	110	24	−35	405	−77	−99	500	180	86	560	0,7	0,6	12%
P5	458	120	23	46	NN	NN	NN	190	51	−68	300	231	151	58%
P6	308	120	23	−24	110	39	16	130	48	20	150	26	14	15%
P7	97	76	1	16	73	25	19	73	−10	−10	88	0,8	−0,9	21%
P8	76	110	11	41	150	54	−29	180	62	480	190	2	−1	6%
P9	67	57	−15	−21	65	−19	16	100	33	23	110	−8	−16	10%
P10	10	51	−12	25	27	−6	−8	63	−9	−7	96	19	4	52%
P11	451	110	5	40	210	410	290	160	47	43	200	0,9	0,7	25%
P12	185	99	−15	36	110	25	−25	110	25	24	130	4	−1	18%
P13	176	130	10	49	537	−131	−58	550	130	61	710	−89	−105	29%
P14	165	205	82	52	160	47	48	170	58	48	200	0,4	0,2	18%

**Figure 5:**
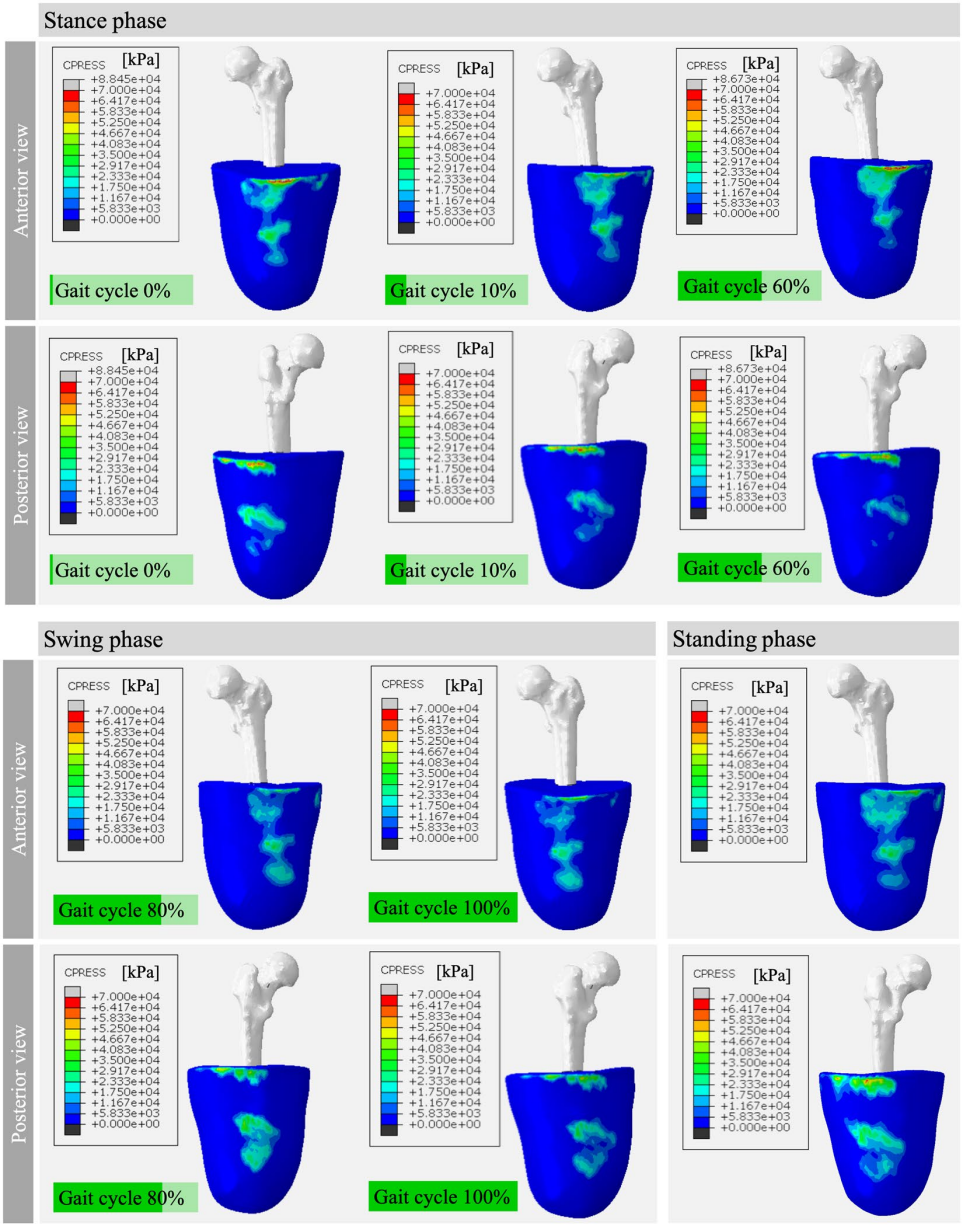
Patient 7 CPRESS distribution during the gait cycle.

By adding the dynamic load to the model, an increase of up to 23% ± 19% was observed in the value of the maximum pressure that occurs at the interaction of the socket and the residual limb. Figure 5 shows the results for reference patient (*P*7) and how the zones and stress distribution changed during the gait cycle.

Although it was expected to find higher stresses at the proximal regions (*i.e*., the edge of the soft tissues reconstruction) as a result of the socket design, there were also stress concentrators due to the partial modelling of the residual limb. When a stress concentrator was causing the maximum stress value rather than what was physically expected due to socket design, the maximum limit value was reduced until reaching a reasonable stress distribution. The reset magnitude was the peak stress value reported in this study. For the patient shown in Fig. 5, there was no need to reset the maximum stress limit.

Table 3 shows the areas where each patient’s model exhibited the greatest pressures (according to Fig. 4) for the standing loading condition and the time during the gait cycle where the peak pressure was identified (columns two and three respectively). The table also indicates what changed from one loading condition to the other based on visual inspection (*e.g*., if the area where the maximum values were located enlarges or contracts) in column four. Column five indicates the moment of the gait cycle at which peak pressure was identified.

The maximum values and their locations were compared to the outcomes of a comfort test carried out during the experiments. The responses in the test indicate whether the patient feels comfortable or not using the prosthesis. In the simulation, if the largest pressure is located in a sensitive area (defined in Fig. 4), it could be assumed that the patient did not feel comfortable. On the contrary, if the largest pressure was located in a tolerant zone (defined in Fig. 4), the patient might feel comfortable with the prosthesis. The comfort test results are presented in Table 3 (column nine). Columns seven and eight indicate whether the maximum stresses are located at a tolerant zone or not during the standing and gait cycle phases respectively.

**Table 3:** Zones distribution, changes and period of maximum pressure at the standing and gait cycle phases, comfort perception and models’ interpretation.

Patient	Standing	Gait cycle	Variations between standing and gait cycle	Gait cycle phase	Soft tissues height	Are the maximum stresses located at a tolerant zone?		Does the amputee feel comfortable wearing his/her prosthesis?
						Standing	Gait cycle	
P1	1 (proximal), 5 and 6 (posterior)	1 (proximal), 5 and 6 (posterior)	The area enlarges and the stresses distribution changes within the zone	Midstance	Near the minor trochanter	YES	YES	YES
P2	1 (proximal), 2 and 6 (lateral)	1 (proximal), 2 and 6 (lateral)	The area enlarges	Initial contact	Near the minor trochanter	NO	NO	NO
P3	1 (above the bone) and 6 (posterior)	1 (above the bone) and 6 (posterior)	Both area and stresses distribution remain similar	Terminal swing	Near the minor trochanter	YES	YES	YES
P4	1 (above the bone) and 6 (lateral)	1 (above the bone) and 6 (lateral)	The area enlarges	Initial contact	Second femoral third	YES	YES	NO
P5	1 (above the bone) and 6 (lateral)	1 (above the bone) and 6 (lateral)	The area enlarges and the stresses distribution changes within the zone	Midstance	Second femoral third	YES	YES	YES
P6	1, 2 and 6 lateral	1, 2 and 6 lateral	The area enlarges	Terminal stance	Near the minor trochanter	NO	NO	NO
P7	1 (above the bone) and 6 (posterior)	1 (above the bone) and 6 (posterior)	The area enlarges	Initial contact	Second femoral third	YES	YES	YES
P8	1 (above the bone) and 6 (posterior)	1 (above the bone) and 6 (posterior)	The area enlarges	Initial contact	Near the minor trochanter	YES	YES	YES
P9	1 (proximal), 2 and 6 (posterior)	2 and 6 posterior	The area enlarges, especially over zone 2	Midstance	First femoral third	NO	NO	NO
P10	1 proximal	1 proximal	Both area and stresses distribution remain similar	Pre-swing	Second femoral third	YES	YES	YES
P11	1 (above the bone) and 6 (posterior)	1 (above the bone) and 6 (posterior)	The area enlarges	Initial contact	Near the minor trochanter	YES	YES	YES
P12	6 (posterior)	1 and 6 (posterior)	The area enlarges	Initial contact	Second femoral third	YES	YES	YES
P13	1 (above the bone) and 6 (posterior)	1 (above the bone) and 6 (posterior)	Both area and stresses distribution remain similar	Loading response	Near the minor trochanter	YES	YES	YES
P14	1 (proximal), 2 and 6 (lateral)	2, 6 (lateral) and 7	The area enlarges	Initial contact	Near the minor trochanter	NO	NO	NO

It should be pointed out that there was variation not only among the geometry of the 14 residual limbs, but also in the distance between the greater trochanter and the proximal surface of the soft tissues used in the models. For some patients, the reconstruction of the soft tissues was performed up to the lesser trochanter of the femur. However, for others, the reconstruction was conducted only until the proximal third of the residual femur or even lower (see column six, Table 3). Furthermore, the length of the residual bone for each individual varied significantly.

## Discussion

This study focused on the differences in residual-limb stress distribution, when different types of loads are applied (static vs dynamic) to a FEA of transfemoral amputees, especially on maximum pressure distribution. The study is based on the fact that excessive pressure causes multiple inconveniences that delay the rehabilitation of patients and prevent them from feeling comfortable with their prosthesis. Most common inconveniences include pain, local blood circulation disruption, erosion of the distal residual limb followed by ulceration, residuum edema syndrome, intertrigo, chronic ulcers^13^, and skin breakdown^40^. The study included 14 patients and included the evaluation of the effect of geometrical differences and amputation characteristics on the stress distribution by adding dynamic loads.

As reported in Dickinson *et al*.^1^, there are not many studies using dynamic models with transfemoral amputees so the comparison with other authors is not extensive. However, the peak pressure values obtained in this study (ranging from 88 to 710 *kPa*) correspond in order of magnitude with those reported in Zhang *et al*.^41^, who also added dynamics loads due to gait cycle (*i.e*., heel strike, midstance, terminal stance in their simulation). Their maximum pressure value was 119 *kPa* at the ischial bearing area. Also, Mu *et al*.^42^ developed a 3D FEA model whose results were compared to Mflex Sensor Distributing System measurements. The maximum stress in the FEA model — with a value of 258 *kPa* — was found at the distal end of the residual limb and it was compared to the maximum stress of 259 *kPa* measured by the sensor system. Nonetheless, it is important to note that peak pressures in these studies were located at the distal end of the residual limb instead of closer to the ischium, as expected in an ischial containment socket and found in this study. Additionally, when looking at the reaction force magnitudes at the acetabulum, at the end of the donning phase, values are in agreement with the order of magnitude of the loads applied at the socket base and the ones reported by other authors^3,41^, which supports the accuracy of the simulations.

Although the lack of the pelvis bone does not accurately reflect what is happening at the ischial containment socket by supporting the loads at this region^3^, when comparing the model proposed by van Heesewijk *et al*.^3^ with this model, this dynamic model can compensate to some extent by an adequate reconstruction of the residual limb (*i.e*., no supine position of the subject which implies compressed soft tissues close to the pelvis) and the socket (*i.e*., rectified geometry, not sharing the internal geometry with the external geometry of the soft tissues). The correct positioning of the parts using the trochanter bone as a reference led to a model closer to the real condition from the start (*i.e*., donning phase). This can be observed in the cation of the peak stresses close to the ischium and not the distal end of the residual limb. Nonetheless, future work including the pelvis is proposed, where the contact condition.between the femur and the pelvis is also taken into account, together with a reconstruction of the soft tissues up to the gluteal region. The addition of this anatomical region will improve the interpretation of the residual limb stresses due to the external moments applied to the distal of the socket. Moreover, the inclusion of the gluteal region, with no deformation, could be possible thanks to the reliable results found scanning anatomical members directly^43^.

Finally, the escalation of the gait cycle moment curves will also be improved by directly including the residuum length in the calculation, so the gait cycle effect on the applied moments is considered. Although the inclusion of the amputee’ specific curves can lead to models closer to reality by incorporating the change in cadence and reaction forces during the gait cycle (both related to BMI), it will introduce misleading results by increasing its variability, since the curves are affected by the individual particularities (*i.e*., pathologic gait).

Except for patient *P*2, all amputees showed greater magnitudes of the maximum value when modelling the loads corresponding to the gait cycle versus the standing phase values. All of them had an increase or redistribution of the areas affected by the maximum pressure values. Besides, excluding patient *P*3, the maximum pressure values occurred during the stance phase of the gait cycle. This is consistent with the large flexion moment created during the initial contact, due to the location of the ground reaction force anterior to the hip joint. However, no explanation was found for the behaviour of *P*3 stress distribution based on the variables included in the study.

The maximum values encountered during the whole gait cycle were compared against the standing phase for each type of stress. At the moment of maximum pressure, both circumferential and longitudinal peak shear stresses did not match the peak pressure behaviour, which was always greater during the gait cycle (as can be seen in the reported data, the shear stresses were greater during the standing phase). Additionally, when analysing the shear stresses present during the gait cycle, the maximum circumferential shear stresses were lower for 10 subjects, while seven subjects also presented lower longitudinal shear stresses at this phase. This behaviour could be explained by the gait cycle relieving the shear strains due to the application of forces and moments in all three axes, liberating the pre-stressed condition created during the donning phase. Physically, this can be interpreted as pistoning happening between the socket and the residual limb. Consistently, during the stan.ing phase, this condition is intensified due to the load applied.

All amputees that presented a change of more than 20% between the standing and walking phase had a *BMI*_*E*_ that exceeded 25 (*i.e*., they were overweight or obese). However, five amputees showed a differen between 5% and 20%. in the maximum pressure value while having a *BMI*_*E*_ higher than 25. Conversely, amputees with a *BMI*_*E*_ lower than 25 did not have a difference of more than 20% in stresses values. Hence, it can be presumed that *BMI*_*E*_ is one of the variables influencing the maximum stresses magnitudes. Further research, including other variables, is required to explore when the force and moment curves used during the gait cycle are not dependant.

For the comparison with the comfort perceived by the patients, except for patient *P*4, the hypothesis was fulfilled. When the highest pressure was located at the sensitive areas, it would indicate that the patient does not feel comfortable. If it is located in the tolerant zone, the patient might feel comfortable with the prosthesis. Apart from these regions, the pressure comfort threshold for each patient should also be taken into account during the comfort analysis. This threshold may indicate how much pressure the person can stand and may be independent of the region of application. This could explain the disparities in patient *P*4.

It should be noted that for regions 3, 4, 5 and 7 described in Table 3, it was not possible to conduct a complete analysis. For region 3, due to the bonded interaction used between the bone and the residual limb, it was not possible to obtain the value of the pressure on the distal end of the bone. However, this interface is recognized as one of the places where great stresses are present, coinciding with other authors^20^. For region 4, the scar was not reconstructed, so it was not considered. Only regions 5 and 7 were taken into consideration for patients whose residual limb was modelled close to or above the lesser trochanter.

For the atypical results that either did not coincide with the hypothesis or were not in line with what was observed in most amputees, no explanation could be found beyond the marked geometrical differences, or based on the variables involved in this study. By including dynamic loads in the FEA for transfemoral amputees, specifically the complete gait cycle, it was possible to provide a complete view of how stresses and the areas affected at each moment change, giving practical values that will support better decisions in patients’ rehabilitation. Especially, FEA could provide quantitative tools to consider factors such as comfort, which are subjective and difficult to measure. For example, the development of a valid and reliable model that can relate comfort perception to the stresses generated at the residual limb by the strains created through socket design. The model could lead to the quantitative definition of the amount of rectification that amputees can bear according to their comfort threshold.

Likewise, it has been shown that specific solutions are required for numerical models to benefit the quality of life of patients. Computational tools can cover those particularities. For example, in the case of transfemoral amputees, whether it is conservative or not to consider only static loads, the areas in which the greatest stresses are located, and the deviations due to compensation that are present in the different phases of their gait cycle.

Finally, the study concludes that the magnitudes of the maximum values of stresses generally increase in dynamic models. Usually, these values appear during the stance phase and the locations remain the same when changing from static to dynamic condition. However, the peak pressure region can increase in size only or can increase in both magnitude and size. Hence, the residual-limb initial stresses state that is established at the time of designing and manufacturing the socket is crucial, since the location of the maximum stresses induced by the external loads do not change significantly. Although these locations may give evidence about the comfort perceived by the patient, direct measurements should be taken to establish a real comfort pressure threshold.

## Data Availability

Data is available from the corresponding author on reasonable request.
